# Carotid Artery Stenosis Contralateral to Intracranial Large Vessel Occlusion: An Independent Predictor of Unfavorable Clinical Outcome After Mechanical Thrombectomy

**DOI:** 10.3389/fneur.2018.00437

**Published:** 2018-06-12

**Authors:** Volker Maus, Nuran Abdullayev, Henrik Sack, Jan Borggrefe, Anastasios Mpotsaris, Daniel Behme

**Affiliations:** ^1^Department of Diagnostic and Interventional Neuroradiology University Medical Center, Göttingen, Germany; ^2^Department of Diagnostic and Interventional Radiology University Hospital, Cologne, Germany; ^3^Department of Diagnostic and Interventional Neuroradiology University Hospital, Aachen, Germany

**Keywords:** acute ischemic stroke, large vessel occlusion, mechanical thrombectomy, carotid artery stenosis, carotid artery stenting

## Abstract

**Background:** Clinical outcome in patients undergoing mechanical thrombectomy (MT) due to intracranial large vessel occlusion (LVO) in the anterior circulation is influenced by several factors. The impact of a concomitant extracranial carotid artery stenosis (CCAS) contralateral to the intracranial lesion remains unclear.

**Methods:** Retrospective analysis of 392 consecutive patients treated with MT due to intracranial LVO in the anterior circulation in two comprehensive stroke centers between 2014 and 2017. Clinical (including demographics and NIHSS), imaging (including angiographic evaluation of CCAS via NASCET criteria), and procedural data were evaluated. Primary endpoint was an unfavorable clinical outcome defined as modified Rankin Scale 3–6 at 90 days.

**Results:** In 27/392 patients (7%) pre-interventional imaging exhibited a CCAS (>50%) contralateral to the intracranial lesion compared to 365 patients without relevant stenosis. Median baseline NIHSS, procedural timings, and reperfusion success did not differ between groups. Median volume of the final infarct core was larger in CCAS patients (176 cm^3^, IQR 32-213 vs. 11 cm^3^, 1-65; *p* < 0.001). At 90 days, unfavorable outcome was documented in 25/27 CCAS patients (93%) vs. 211/326 (65%; *p* = 0.003) with a mortality of 63 vs. 19% (*p* = 0.001), respectively. Presence of CCAS was associated with an unfavorable outcome at 90 days independent of age and baseline NIHSS in multivariate logistic regression (OR 2.2, CI 1.1-4.7; *p* < 0.05).

**Conclusion:** For patients undergoing MT due to intracranial vessel occlusion in the anterior circulation, the presence of a contralateral CCAS >50% is a predictor of unfavorable clinical outcome at 90 days.

## Introduction

Acute ischemic stroke (AIS) often results in a high rate of functional dependency and mortality (1). Several factors affect the clinical outcome in patients suffering from intracranial large vessel occlusion (LVO), for example age, clinical severity of stroke expressed by the National Institutes of Health Stroke Scale (NIHSS) on admission, completeness of occlusion, angiographic reperfusion result, and concomitant application of intravenous thrombolysis (IVT) (1–5). According to current guidelines, mechanical thrombectomy (MT) is the standard therapy for LVO, with concomitant IVT whenever possible because swift and complete reperfusion is the hallmark for a successful treatment (6). Another factor influencing functional dependency is the presence of potentially salvageable brain tissue (“penumbra”) in relation to core infarct size, which depends on the collateral status (7). A collateral flow of the affected territory is often supported by cross-over flow via the contralateral carotid artery (8). However, a fraction of patients also suffer from contralateral carotid artery disease as patients frequently have atherosclerotic involvement of multiple intracranial and/or extracranial vessels (9). The effectiveness of collateral circulation under these circumstances remains elusive.

In a previous study, we observed that for patients undergoing MT and carotid artery stenting due to acute anterior tandem occlusion, the presence of a contralateral carotid artery stenosis >50% is an independent predictor of unfavorable clinical outcome, which presumably was a consequence of poorer collateral flow to the affected tissue (10). In this study, we analyzed the impact of a concomitant extracranial carotid artery stenosis (CCAS) contralateral to an intracranial LVO and hypothesized that the presence of a contralateral CCAS has a negative impact on the clinical outcome at 90 days.

## Materials and methods

### Study design and patient selection

A retrospective analysis of data from two comprehensive stroke centers was performed to identify all consecutive patients who were treated with MT due to intracranial LVO of the anterior cerebral circulation between April 2014 and December 2017. Baseline and angiographic parameters were extracted from prospectively acquired databases. According to the guidelines of the respective local ethics committees, ethic approval was given for this anonymous retrospective study, which was conducted in accordance to the Declaration of Helsinki.

Inclusion criteria for endovascular therapy of an intracranial LVO in the anterior circulation changed over time. Initially, MT was performed for middle cerebral artery stroke by using the one-third rule based on the exclusion criteria of the SWIFT and TREVO-2 trials (11–13). With growing experience and subsequent research, the presence of pre-interventional, multi-detector computed tomography (MDCT) including non-contrast CT and single-phase CT angiography (MDCTA) or flat-detector CT (FDCT) and multiphase FDCTA in cases of one-stop management with evidence of an arterial LVO were required (14). Based on the MR CLEAN trial, no general limitations existed since 2015 with regard to baseline Alberta Stroke Program Early CT Score (ASPECTS) and NIHSS on admission or procedural characteristics including the use of different equipment and thrombectomy techniques, which were left to the attending neuroradiologist's discretion (15). Missing of pre-treatment imaging in the picture archiving system, e.g., in “drip-and-ship” situations, led to exclusion of this cases. All patients treated due to isolated occlusion of anterior cerebral artery and with an unknown onset of symptoms were excluded. Patients treated with MT and acute carotid artery stenting due to tandem occlusions were excluded as this cohort was analyzed in a prior study (10). According to neurological guidelines, patients received IVT whenever possible. Angiographic results were locally graded by a neuroradiologist according to the modified Thrombolysis in Cerebral Infarction score (mTICI). Symptomatic intracranial hemorrhage (sICH) was defined as any extravascular blood in the brain or within the cranium that was associated with clinical deterioration, as defined by an increase of ≥4 points in the NIHSS score (16). Final infarct volume was assessed with ABC/2 method (17). Clinical outcome was assessed with the modified Rankin Scale (mRS) at discharge and after 90 days by a certified stroke neurologist in each center. The primary endpoint was an unfavorable clinical outcome 90 days after treatment defined as mRS 3–6. Secondary endpoints were final infarct volume and post-interventional occurrence of symptomatic intracranial hemorrhage (sICH).

### Angiographic analysis of carotid artery stenosis

Stenosis of the contralateral carotid artery was established on CTA and mainly obtained from axial images using the North American Symptomatic Carotid Endarterectomy Trial equation (NASCET) (18). Measurements were performed by a neuroradiologist at each center. For MDCTA, the centers utilized a bolus trigger technique with the region of interest in the ascending aorta; for FDCTA, the scan was manually started if the cavernous segment of the internal carotid artery (ICA) was flushed with contrast agent. Standard axial slices of 1.25 or 0.6 mm thickness were obtained and maximum intensity projections were reconstructed according to local standard operating procedures. Distal measurements were taken at a disease-free portion of the ICA no fewer than 2 mm distal to luminal stenosis. In case of a horizontal or tortuous course of the vessel or a very short stenosis, maximum intensity projections and reconstructions were consulted (19). A luminal stenosis of >50% was defined as a relevant CCAS (10). The amount of contrast agent was 1.2–1.5 mL/kg body mass and minimum flow rate was 4.5 mL/s. Different types of CT systems were used (MDCT: Brilliance iCT [256-row], Philips, Hamburg, Germany and Somatom Definition AS+ [128-row], Siemens Healthcare, Erlangen, Germany; FDCT: Artis Q, Siemens Healthcare).

### Statistical analysis

Statistical analysis was performed using JMP 12.0 Software (SAS Institute, Cary, NC, USA). Descriptive statistics of normally distributed variables are summarized as mean ± standard deviation, otherwise as median ± interquartile range (IQR). Wilcoxon test and 2-tailed Fisher's exact test were performed for assessing statistical differences between groups. Multivariate logistic regression with age and admission NIHSS was used to determine the independence of CCAS as predictor of unfavorable clinical outcome as age and stroke severity are known to be the most important determinants of stroke outcomes (20). Statistical significance was defined as *p* ≤ 0.05.

## Results

**Figure 1:**
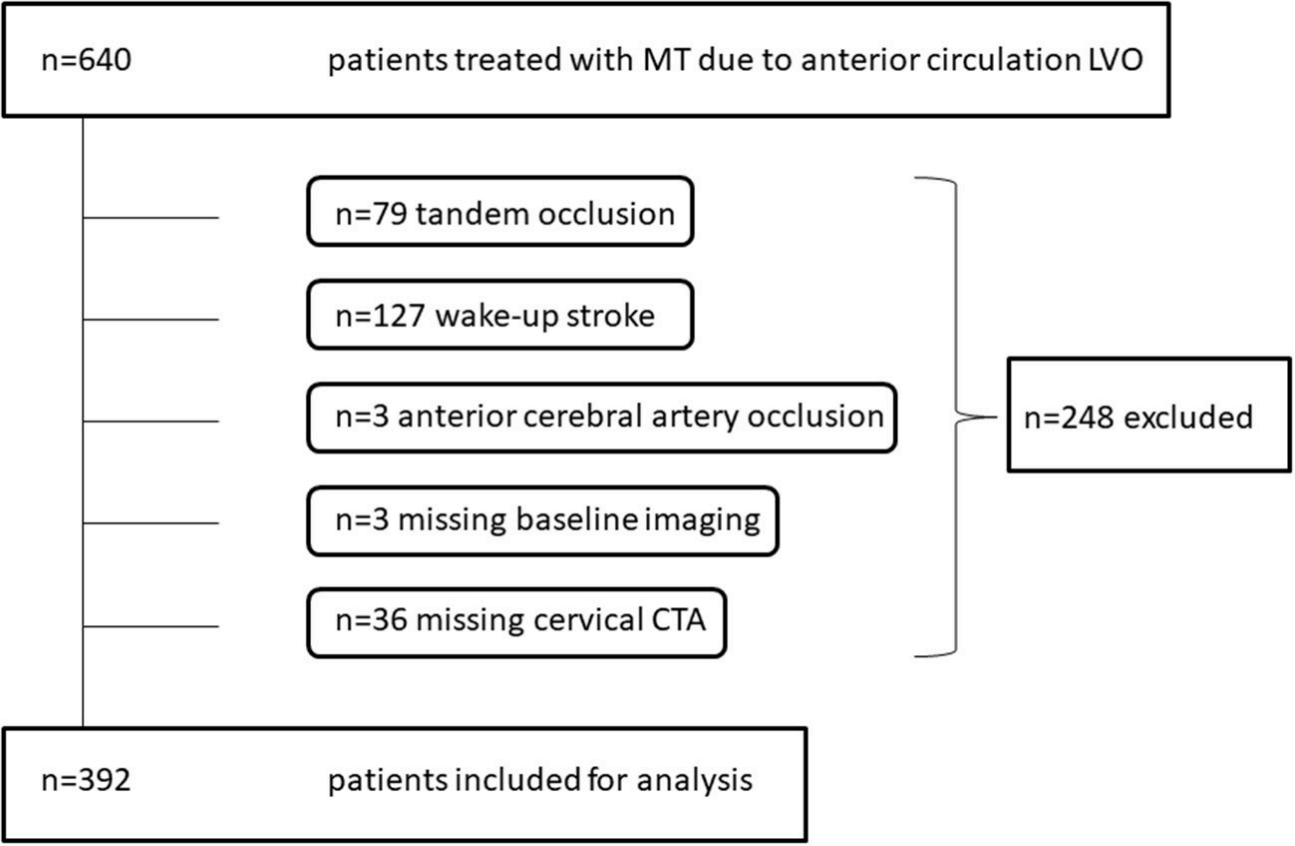
Flow of patients through this study.

Six hundred forty patients were treated with MT due to intracranial LVO of the anterior circulation. Of those, 392 patients met inclusion criteria during a time period of 69 months (Figure 1). Mean age was 73 ± 13 years. One hundred seventy-five patients (45%) were male. Median baseline NIHSS was 15 (IQR 10-20). Intracranial occlusion sites were as follows: ICA-T in 44/392 (11%), M1 segment of middle cerebral artery in 301/ 392 (77%), and M2 in 47/392 (12%). Median groin puncture to reperfusion time was 45 (IQR 30–72) min with a successful reperfusion rate (mTICI ≥2b) of 84%. Ninety days' follow-up data were available for 353 patients (90%). Of those, 117 patients (33%) showed a favorable clinical outcome (mRS ≤ 2) and overall mortality was 23%.

Twenty-seven (7%) individuals exhibited a CCAS contralateral to an intracranial LVO with a luminal stenosis >50 and >70% in 7 (2%) cases. Two patients (0.5%) had bilateral carotid artery stenosis. Fifteen patients (56%) with CCAS were male (Table 1). CCAS patients tended to be older than patients without carotid artery disease with a mean difference of 3.5 years (*p* = 0.081). Clinical severity on admission based on the median NIHSS did not differ between groups (16, IQR 10-22 vs. 15, IQR 10-19). IVT was applied in 16/27 CCAS patients (59%). Neither median groin-puncture to reperfusion (51, IQR 39–72 vs. 45, IQR 29–72 min) nor onset to reperfusion time (242, IQR 198–284 vs. 234, IQR 185–300 min) were significantly different. Reperfusion was successful in 25/27 patients (93%) vs. 303/365 (83%, *p* = 0.773), respectively. SICH occurred in 4/27 patients (15%) vs. 21/363 (6%, *p* = 0.084). Median volume of the final infarct core on post-interventional CT at discharge was larger in CCAS patients (176, IQR 32-213 vs. 11 cm^3^, IQR 1-65; p = 0.001).

**Table 1:** Characteristics and outcome of patients with carotid artery stenosis contralateral to an intracranial large vessel occlusion.

	**Non-CCAS**	**CCAS >50%**	***p*-value**
No. of patients	365	27	
**BASELINE CHARACTERISTICS**			
Age, mean ± sd	73 ± 13	77 ± 8	0.081
Men, n (%)	160 (44)	15 (56)	0.490
Hypertension, n (%)	271 (74)	22 (82)	0.766
Coronary artery disease, n (%)	98 (27)	14 (52)	0.075
PAOD, n (%)	25 (7)	4 (15)	0.255
Diabetes mellitus, n (%)	79 (22)	11 (41)	0.125
Obesity, n (%)	78 (21)	4 (15)	0.628
Smoker, n (%)	56 (15)	6 (22)	0.429
Chronic kidney disease, n (%)	59 (16)	7 (26)	0.307
ASPECTS pre-interventional, median (IQR)	9 (8-10)	9 (7-9)	1.0
**CLINICAL CHARACTERISTICS**			
NIHSS baseline, median (IQR)	15 (10–19)	16 (10–22)	1.0
IVT, n (%)	242 (66)	16 (59)	0.870
**INTRACRANIAL OCCLUSION SITE**			
ICA-T, n (%)	39 (11)	5 (19)	0.353
M1, n (%)	283 (77)	18 (67)	0.756
M2, n (%)	43 (12)	4 (15)	0.761
**PROCEDURAL DATA**			
Onset to Imaging [minutes], median (IQR)	112 (48–131)	115 (57–141)	1.0
Onset to Groin puncture [minutes], median (IQR)	180 (137–225)	184 (155–219)	1.0
Onset to Reperfusion [minutes], median (IQR)	234 (185–300)	242 (198–284)	1.0
Groin puncture to Reperfusion [minutes], median (IQR)	45 (29–72)	51 (39–72)	1.0
Successful Reperfusion, mTICI ≥2b, n (%)	303 (83)	25 (93)	0.773
**CLINICAL OUTCOME**			
sICH, n (%)	21 (6)	4 (15)	0.084
mRS 3–6 at 90 days	211 (65%)	25 (93)	**0.003**
Mortality, n (%)	63 (19)	17 (63)	**0.001**
Final infarct volume, median (IQR)	11 (1–65)	176 (32–213)	**<0.001**

*CCAS, concomitant extracranial carotid artery stenosis; sd, standard deviation; PAOD, Peripheral artery occlusive disease; NIHSS, National Institutes of Health Stroke Scale; IVT, intravenous thrombolysis; ASPECTS, Alberta Stroke Program Early CT Score; IQR, interquartile range; mTICI, modified Thrombolysis in Cerebral Infarction; sICH, symptomatic intracranial hemorrhage; mRS, modified Rankin Scale. Bold values indicate the statistical significant*.

At 90 days, unfavorable outcome was present in 25 out of 27 CCAS patients (93%) vs. 211 out of 326 (65%; *p* = 0.003) with a mortality of 63 vs. 19% (*p* = 0.001), respectively. In logistic regression analysis, the presence of a CCAS contralateral to an intracranial LVO was associated with an unfavorable clinical outcome when adjusted for age and baseline NIHSS (OR 2.2, CI 1.1-4.7; *p* < 0.05, Table 2).

**Table 2:** Odds ratios for association of carotid artery stenosis contralateral to an intracranial large vessel occlusion, age, baseline NIHSS, and selected cardiovascular risk factors with poor clinical outcome after 90 days (mRS 3–6).

	**Univariate models**	**Multivariate models**
CCAS	2.6 (1.2–5.4)^**^	2.2 (1.1–4.7)^*^
Age	2.0 (1.6–2.5)^***^	1.8 (1.4–2.4)^***^
NIHSS	2.5 (1.9–3.3)^***^	2.6 (2.0–3.3)^***^
Coronary artery disease	1.6 (1.2–2.1)^**^	1.5 (1.1–2.1)^*^
Chronic kidney disease	1.5 (1.1–2.1)^*^	1.2 (0.9–1.7)^#^

* *p < 0.05*,

** *p < 0.01*,

*** *p < 0.001*,

# *not significant*.

## Discussion

Since randomized controlled trials proved superiority of MT compared to best medical treatment alone, endovascular therapy in patients suffering from AIS due to LVO is nowadays state of the art (21). Several factors are known to predict clinical outcome such as patient age, clinical severity of stroke, completeness of occlusion, angiographic reperfusion result, and concomitant IVT (1–5). The potential role of an ICA stenosis contralateral to intracranial LVO and the subsequent hemodynamic alterations is poorly understood. In our study, we demonstrated that the presence of a contralateral CCAS in AIS has a negative impact on outcome at 90 days.

There is no sufficient data with regard to contralateral CCAS in the setting of AIS. However, there is limited data in the role of CCAS in patients who underwent carotid endarterectomy (CEA) (9, 22). AbuRhama et al. report a benign course of carotid artery occlusion contralateral to CEA after surgery with a late stroke rate of 4% at 5 years of the contralateral site (22). Contradictory findings were reported from Da Silva et al. who found an ICA occlusion contralateral to CEA be associated with an increased risk of death and stroke at 30 days (5.6% vs. 2.4) (9). These studies, however, focused on the effect of CEA when contralateral carotid artery was occluded and observed perioperative complications and new strokes in the postoperative period.

The present study is a subsequent study to our prior observation that a contralateral CCAS in acute tandem occlusions has a negative impact on clinical outcome (10). Thus, patients treated with MT and acute carotid artery stenting due to tandem occlusions were excluded from this study with the consequence that the number of patients with bilateral carotid artery disease was underrepresented. However, occlusive carotid artery disease is often asymmetric with hemodynamic significant stenosis limited to one side (23). In the current analysis, CCAS contralateral to an intracranial LVO showed a higher rate of an unfavorable outcome (93 vs. 65%) with an increased mortality (63 vs. 19%). A possible explanation might be that CCAS patients exhibited a larger final infarct volume, which was recently demonstrated to be associated with a worse functional outcome in AIS (24). This might be a consequence of a poor collateral status. Our results are endorsed by a former study, which was the first to report a causal connection between AIS of the anterior circulation and patency of the contralateral carotid artery (25). The authors demonstrated that mortality was higher when contralateral CCAS >50% was present and ipsilateral carotid artery was patent as this was the case in our cohort. An explanation for the unfavorable outcome and the increased infarct volume could be that the patent ipsilateral carotid artery may already be compensating the chronic CCAS on the other side by contributing to the collateral circulation in the contralateral hemisphere and thus collateral supply could not have been developed in the ipsilateral hemisphere over time (25).

In our opinion, the similar baseline ASPECTS between CCAS and non-CCAS patients does not disagree with our hypothesis of poor collateral status as at the early time point of initial imaging precludes significant differences with regard to early ischemic changes in non-contrast head CT. Even in MR CLEAN, the median baseline ASPECTS was 9 in both groups with a median time from stroke onset to randomization of 204 min, which is longer compared to our time from onset to imaging (180 and 184 min, respectively) (15).

Patients with atheromatous cerebrovascular disease often have a distinct cardiovascular risk profile with multifocal atherosclerotic lesions (26). In our study, the history of coronary artery disease (CAD) was a predictor of clinical outcome. This accompanies with a sub-analysis of the European Cooperative Acute Stroke Study (ECASS) I, which detected the presence of CAD as an independent prognostic factor for early progressing stroke (27). However, occurrence of CAD in our patients was documented based on the patient's medical history and it remained unclear whether CAD was symptomatic or treated medically. The development of atherosclerotic lesions depends upon age and (male) sex (28). We found that more than half of the patients presenting with contralateral CCAS were male and tended to be older, but this did not reach statistical significance. Similar to our previous study, the occurrence of sICH did not differ significantly between groups and therefore sICH might not be responsible for the poorer clinical outcome in the CCAS cohort.

Strengths of our study are the large number of consecutive patients. One major limitation could be the retrospective design with a possible selection bias. As poor collateral flow status might be responsible for the unfavorable outcome in CCAS patients, an assessment of collateral score would be desirable. However, the heterogeneity of CTA assessment (single vs. multi-phase) in the centers precludes a distinct evaluation of the collateral status. Angiographic analysis of the carotid artery lesions and final angiogram was self-reported and may be less favorable after core laboratory adjudication. The extent of the final infarct volume might be overestimated due to the chosen formula. Furthermore, the formula might not be adequate for small infarct volumes as the volumes of the non-CCAS patients were very small, however, the differences observed were distinct between the groups.

In conclusion, for patients undergoing MT due to intracranial LVO in the anterior circulation, the presence of a contralateral CCAS >50% is a predictor of unfavorable clinical outcome at 90 days. This study suggests that an assessment of the extracranial vasculature contralateral to the occlusion site should be encouraged during the acute phase of stroke in order to provide useful prognostic information.

## Author contributions

The idea and initiation of the present study was done by AM and DB. The manuscript was written by VM. Data collection was performed by NA and HS. The statistical analysis was executed by JB. Proof reading was conducted by all authors.

### Conflict of interest statement

The authors declare that the research was conducted in the absence of any commercial or financial relationships that could be construed as a potential conflict of interest.
